# Analysis of Prognostic Risk Factors and Establishment of Prognostic Scoring System for Secondary Adult Hemophagocytic Syndrome

**DOI:** 10.3390/curroncol29020097

**Published:** 2022-02-15

**Authors:** Qiaolei Zhang, Youyan Lin, Yejiang Bao, Yuan Jin, Xiujin Ye, Yamin Tan

**Affiliations:** 1 Department of Hematology, Cancer Hospital of the University of Chinese Academy of Sciences (Zhejiang Cancer Hospital), Hangzhou 310022, China; zhangql2007@126.com (Q.Z.); fangbingmu@126.com (Y.L.); weizhangyuezj1@126.com (Y.B.); lixueyingzj1@126.com (Y.J.); 2 Institute of Cancer and Basic Medicine (IBMC), Chinese Academy of Sciences, Hangzhou 310000, China; 3 Department of Hematology, The First Affiliated Hospital of Medical School of Zhejiang University, Hangzhou 310058, China; wuyingzj1@126.com

**Keywords:** hemophagocytic syndrome, risk factors, prognosis, scoring system

## Abstract

Introduction: The objective of this paper is to identify the prognostic risk factors of secondary adult hemophagocytic syndrome (HLH) in hospitalized patients and establish a simple and convenient prognostic scoring system. Method:We reviewed 162 adult patients secondary with HLH treated in Zhejiang Cancer Hospital and the First Affiliated Hospital of Medical College of Zhejiang University from January 2014 to December 2018 were enrolled to form the test group; from January 2019 to February 2021, 162 adult patients in the hospitals constituted the validation group. The HLH prognosis scoring system was constructed according to the risk factors, and the patients were divided into three risk groups: low risk, medium risk, and high risk. The scoring system was verified by Kaplan–Meier method and log rank test survival analysis. The discrimination ability was evaluated according to the receiver operating characteristic (ROC) curve. Results: Univariate and multivariate analysis showed that the independent risk factors for the prognosis of HLH were male sex, activated partial prothrombin time (APTT) greater than 36 s, lactate dehydrogenase (LDH) greater than 1000 U/L, and C-reactive protein (CRP) greater than 100 mg/L. The area under the ROC curve was 0.754 (95% Cl: 0.678–0.829). The patients were divided into a low-risk group (0–1), a medium-risk group (2–4), and a high-risk group (5–6). The 5-year overall survival (OS) rate were 87.5%, 41.8% and 12.8%, respectively (*p* < 0.001). The area under ROC curve was 0.736 (95% Cl: 0.660–0.813) in the validation group, and the 2-year OS of patients in low-risk, medium-risk and high-risk groups were 88.0%, 45.1% and 16.7%, respectively (*p* < 0.001). Conclusion:The new prognostic scoring system can accurately predict the prognosis of secondary adult HLH and can further provide basis for the accurate treatment of secondary adult HLH.

## 1. Introduction

Hemophagocytic syndrome, also known as hemophagocytic lymphohistiocytosis (HLH), is a clinical syndrome of immune overactivation, particularly of lymphocytes and histiocytes, with resultant hypercytokinemia [1]. HLH disease may be associated with specific genetic and/or environmental causes. HLH disease mimics are disorders that resemble HLH syndrome but are caused by other conditions. Historically, HLH has been divided into a primary form and secondary forms. Primary HLH is a heritable disease conferred by highly penetrant genetic mutations/variations impacting cytolytic functions, lymphocyte survival, or inflammasome activation. In contrast, secondary HLH is driven primarily by acquired factors such as chronic inflammation, infection, or malignancy. Uncontrolled and harmful immune activation results in excessive inflammation and tissue destruction. Activated T cells and macrophages secrete high levels of inflammatory cytokines such as IFN-γ, IL-12, IL-18, and TNFα. Genetic defects play a major role in childhood HLH and are increasingly found in adult cases. HLH usually presents as an acute or subacute febrile illness associated with multiple organ involvement; most patients with HLH are acutely ill with multiorgan involvement, cytopenias, liver function abnormalities, and neurologic symptoms. It has complex etiology, lack of specificity in clinical manifestations, and high mortality. It is a serious life-threatening immune disorder [2]. In the treatment process of HLH patients, the clinical staging system is very important to predict the prognosis of patients. At present, there are few clinical prediction models for the prognosis of secondary HLH. This study aims to build a clinical prediction model for the prognosis of secondary HLH and explore its prediction value by screening the risk factors related to the prognosis of secondary HLH.

## 2. Methods

### 2.1. Patients

Retrospective analysis of 324 inpatients diagnosed with secondary adult HLH in Zhejiang cancer hospital and the First Affiliated Hospital of Medical College of Zhejiang University from January 2014 to February 2021, including 188 males and 136 females. Two batches were included successively. The first batch of 162 cases from January 2014 to December 2018 constituted the test group, and the second batch of 162 cases from January 2019 to February 2021 constituted the validation group. This study was reviewed by the ethics committee of Zhejiang Cancer Hospital and the First Affiliated Hospital of Medical College of Zhejiang University. All patients signed informed consent.

### 2.2. Diagnostic Criteria

The diagnosis of HLH was based on the HLH-2004 protocol standard (Group 2004) [3] and North American Consortium for Histiocytosis (NACHO) [4]. Five of the following eight diagnostic criteria had to be met: (1) fever; (2) splenomegaly; (3) cytopenia affecting at least two of three lineages in the peripheral blood; (4) hypertriglyceridemia (triglyceride levels ≥265 mg/dL) and/or hypofibrinogenemia (fibrinogen level ≤150 mg/dL); (5) hemophagocytosis in the bone marrow, spleen, or lymph nodes; (6) low or absent natural killer (NK) cell activity; (7) hyperferritinemia (ferritin level ≥500 μg/L); or (8) high levels of sIL-2R (≥2400 U/mL). The diagnosis of malignant lymphoma was based on the 2016 world health organization (WHO) classification.

### 2.3. Laboratory Examination

Data collected from each patient included age, sex, presumed etiology, presence or absence of splenomegaly, primary disease (tumor, connective tissue disease, infected) and laboratory findings (white blood cell (WBC), absolute neutrophil count (ANC), absolute lymphocyte count (ALC), hemoglobin (HGB), platelet (PLT), globulin (GLB), albumin (ALB), aspartate aminotransferase (AST), alanine aminotransferase (ALT), total bilirubin (TB), direct bilirubin (DB), indirect bilirubin (IB), triglycerides (TG), lactate dehydrogenase (LDH), creatinine, urea, prothrombin time (PT), activated partial thromboplastin time (APTT), fibrinogen (Fib), C-reactive protein (CRP), ferritin, human immunodeficiency virus antibody, and percentage of macrophages in the bone marrow).

### 2.4. Therapeutic Regimens

The two groups underwent the following treatments: etoposide + dexamethasone + cyclosporine; dexamethasone + antibiotics; dexamethasone + immunoglobulins; and ECOP or ECHOP regimen (etoposide + dexamethasone + vindesine + cyclophosphamide + nordoxorubicin). Patients with infections were administered antibiotics, including antiviral drugs. Blood transfusion included transfusion of erythrocyte suspension, platelets, fresh plasma, and blood products (human albumin, human prothrombin complex, human fibrinogen).

### 2.5. Follow Up Time and Primary End Point

The main outcome measure was overall survival, that is, from the date of disease diagnosis to the date of death or the last follow-up (28 February 2021).

### 2.6. Statistical Analysis

Statistical analysis was performed using IBM SPSS 26 software. We compared clinical and laboratory data between test and validation groups, survival and death group using Chi square test. The independent risk factors of OS were analyzed by univariate and multivariate Cox risk regression model, grouped according to the independent risk factors, and survival analysis was carried out by Kaplan–Meier method and logrank test to preliminarily verify the value of independent risk factors. Then, the clinical prediction model scoring system was established by assigning independent risk factors. According to the score, the patients were divided into low-risk, medium-risk, and high-risk groups. Kaplan–Meier method and logrank test survival analysis were used to verify the application value of the scoring system, and ROC curve was used to evaluate the model discrimination ability. *p* < 0.05 was statistically significant.

## 3. Results

### 3.1. Patient Characteristics

A total of 324 patients were included in the study. A total of 162 cases were included in the test group, including 97 males (59.8%), with an average age of 51 years. A total of 162 cases were included in the validation group, including 91 males (56.2%), with an average age of 49 years. As of the end point of follow-up, 181 patients died and 143 survived. There was no significant difference in basic values between the two groups (Table 1 and Table 2).

### 3.2. Treatment of HLH Patients

**Figure 1:**
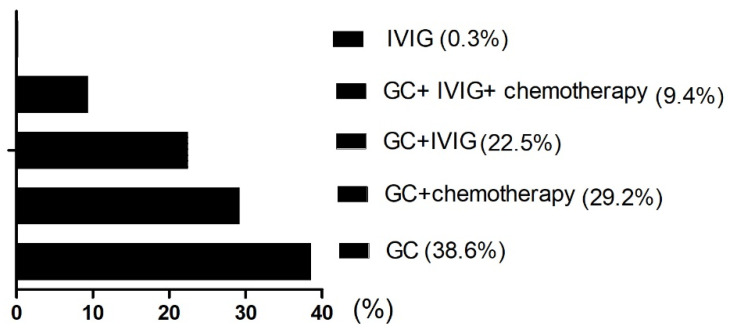
Distribution of five treatment groups in the HLH. GC: Glucocorticoid; IVIG: intravenous immunoglobulin.

One patient received intravenous immunoglobulin (IVIG) alone (0.3%), 132 patients received glucocorticoid (GC) alone (38.6%), 77 patients received GC combined with IVIG (22.5%), 100 patients received GC combined with chemotherapy (29.2%), and 32 patients received GC combined with IVIG and chemotherapy (9.4%; Figure 1).

### 3.3. Comparison of Clinical Characteristics and Laboratory Indexes of Death and Survival Subgroups in the Test Group

Laboratory variables at diagnosis, compared between survivor and death groups, showed statistical significance in male (47.8% vs. 68.8%, *p* = 0.007), age > 60 (20.3% vs. 44.1%, *p* = 0.002), L ≤ 0.5 × 10^9^/L (44.9% vs. 68.8%, *p* = 0.002), Plt ≤ 20 × 10^9^/L (18.8% vs. 44.1%, *p* = 0.001), Fib ≤ 1.5 g/L (53.6% vs. 70.9%, *p* < 0.001), APTT > 36 s (55.1% vs. 86.0%, *p* < 0.001), PT > 13.5 s (53.6% vs. 78.5%, *p* = 0.001), Urea > 8.2 mmol/L (34.8% vs. 55.9%, *p* = 0.008), LDH > 1000 U/L (23.2% vs. 58.1%, *p* < 0.001), ferritin > 10,000 μg/L (34.8% vs. 53.8%, *p* = 0.016), CRP > 100 mg/L (13.0% vs. 31.2%, *p* = 0.007), and connective tissue disease (13.0% vs. 1.1%, *p* = 0.004) (Table 3).

### 3.4. Risk Factors Affecting the Prognosis of Patients with HLH

**Figure 2:**
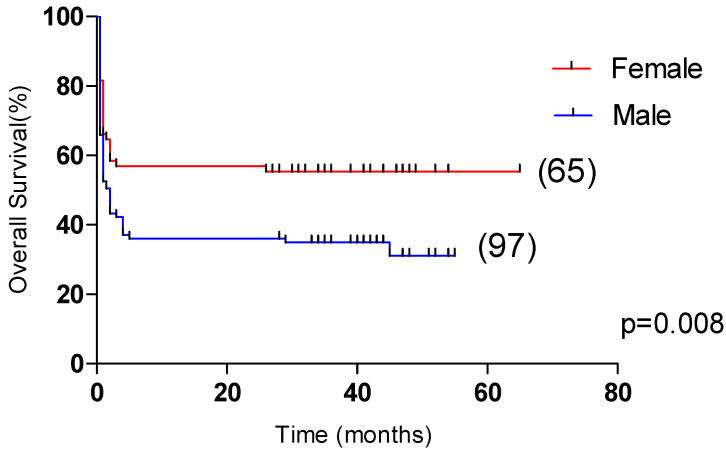
Comparison of overall survival in patients with female group and male group (55.4% vs. 31.1%, *p* = 0.008).

**Figure 3:**
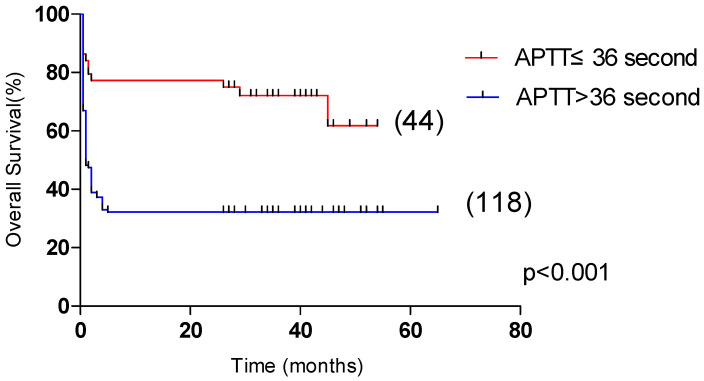
Comparison of overall survival in patients with APTT ≤ 36 s group and APTT > 36 s group (61.8% vs. 32.2%, *p* < 0.001).

**Figure 4:**
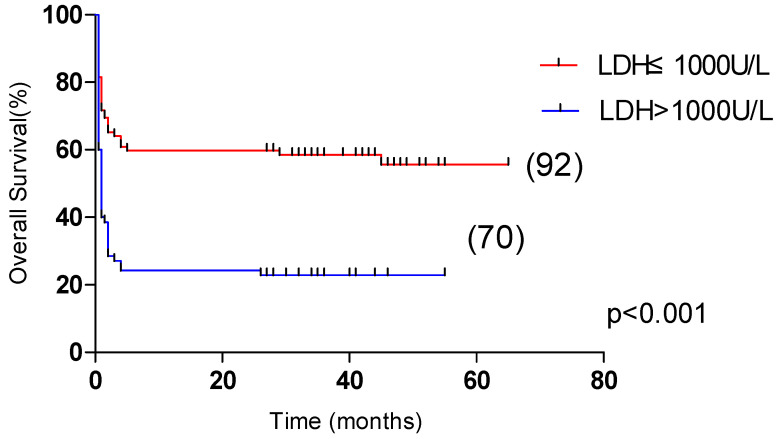
Comparison of overall survival in patients with LDH ≤ 1000 U/L group and LDH > 1000 U/L group (55.6% vs. 22.9%, *p* < 0.001).

**Figure 5:**
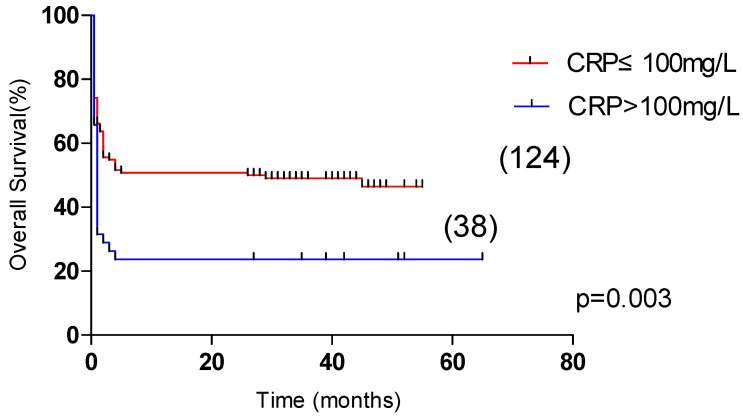
Comparison of overall survival in patients with CRP ≤ 100 mg/L group and CRP > 100 mg/L group (46.5% vs. 23.7%, *p* = 0.003).

Univariate analysis showed that OS was significantly correlated with age >60 years, male, L ≤ 0.5 × 10^9^/L, Plt ≤ 20 × 10^9^/L, APTT > 36 s, PT > 13.5 s, Urea > 8.2 mmol/L, LDH > 1000 U/L, ferritin > 10,000 μg/L, CRP > 100 mg/L were correlated with the overall survival of HLH (*p* < 0.05). Multivariate analysis revealed that male sex, APTT > 36 s, LDH > 1000 U/L and CRP > 100 mg/L, were independent risk factors affecting the overall survival (*p* < 0.05) (Table 4). Among them, the 5-year OS of male and female patients were 31.1% and 55.4%, respectively (*p* < 0.05, Figure 2). The 5-year OS of patients with APTT > 36 s group and APTT ≤ 36 s group were 32.2% and 61.8%, respectively (*p* < 0.05, Figure 3). The 5-year OS of patients with LDH > 1000 U/L group and LDH ≤ 1000 U/L group were 22.9% and 55.6%, respectively (*p* < 0.05, Figure 4). The 5-year OS of patients with CRP > 100 mg/L group and CRP ≤ 100 mg/L group were 23.7% and 46.4%, respectively (*p* < 0.05, Figure 5).

### 3.5. Establishment of Prognostic Scoring System

Based on β coefficient and OR value, assign scores to each risk factor. Male sex, APTT > 36 s, LDH > 1000 U/L, and CRP > 100 mg/L are, respectively, assigned as 1 point, 2 points, 2 points, and 1 point, otherwise it is 0 point, and the total score of the four items is 6 points. The patients were divided into groups, including 12 cases with 0 points, 19 cases with 1 point, 24 cases with 2 points, 36 cases with 3 points, 28 cases with 4 points, 34 cases with 5 points, and 9 cases with 6 points. There was no significant difference in OS between patients with scores of 0 and 1 (*p* > 0.05), between patients with scores of 2, 3, and 4 (*p*> 0.05), and between patients with scores of 5 and 6 (*p*> 0.05). Therefore, we established a simple scoring system according to the score (Table 5). After this, we divided the system into three risk groups: low-risk (0~1 points), medium-risk (2~4 points), and high-risk (5~6 points).

### 3.6. Predictive Value of HLH Scoring System

**Figure 6:**
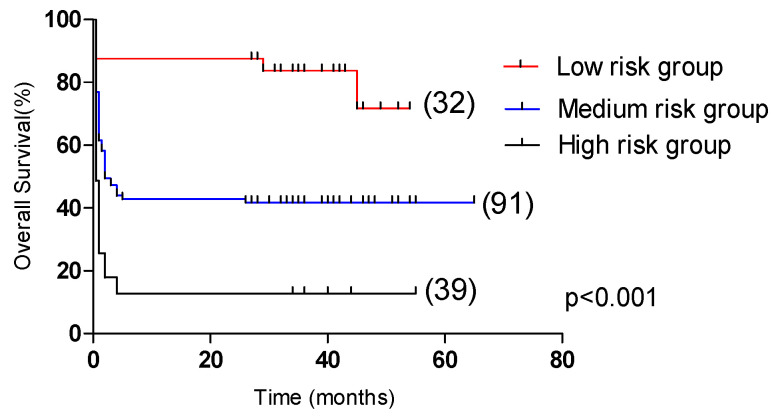
Comparison of overall survival in patients in the low-risk group, medium-risk group, and high-risk group (test group, 71.7% vs. 41.8% vs. 12.8%, *p* < 0.001).

**Figure 7:**
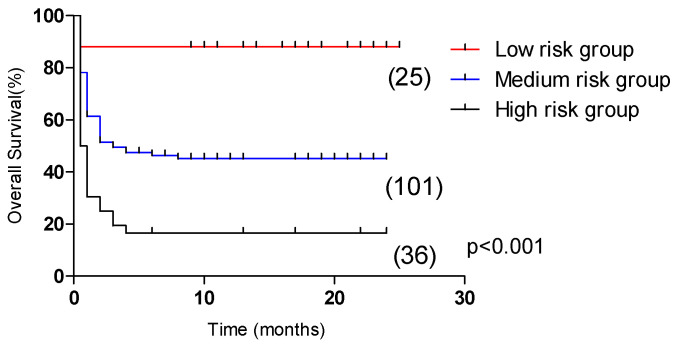
Comparison of overall survival in patients in the low-risk group, medium-risk group, and high-risk group (validation group, 88.0% vs. 45.1% vs. 16.7%, *p* < 0.001).

In the test group, the 5-year OS of the low-risk group (31 cases), medium-risk group (88 cases), and high-risk group (43 cases) had significant statistical significance (*p* < 0.001, Figure 6), which were 71.7%, 41.8% and 12.8%, respectively. In the validation group, the 2-year OS of the low-risk group (25 cases), medium-risk group (101 cases), and high-risk group (36 cases) were significantly statistically significant (*p* < 0.001, Figure 7), which were 88.0%, 45.1% and 16.7%, respectively.

### 3.7. HLH Scoring System Verification

**Figure 8:**
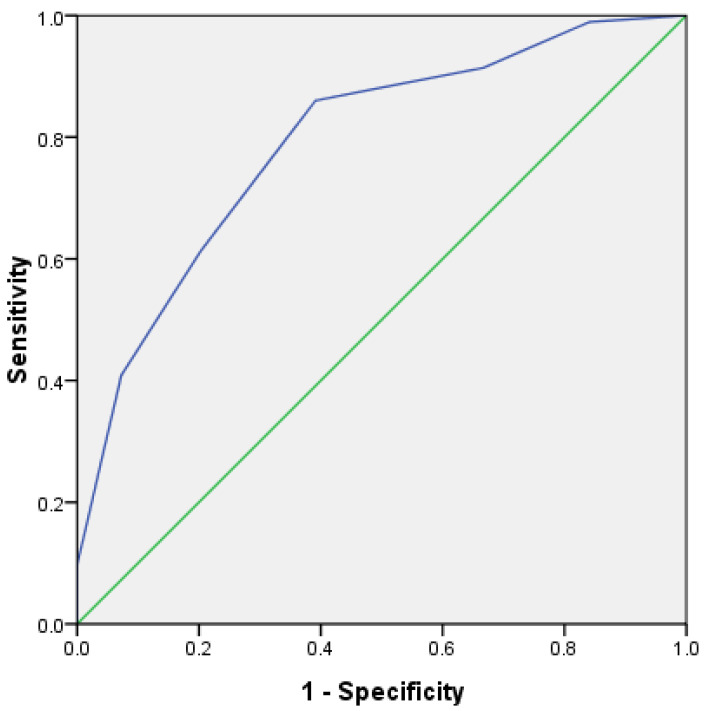
ROC curve for predicting survival risk of HLH (test group).

**Figure 9:**
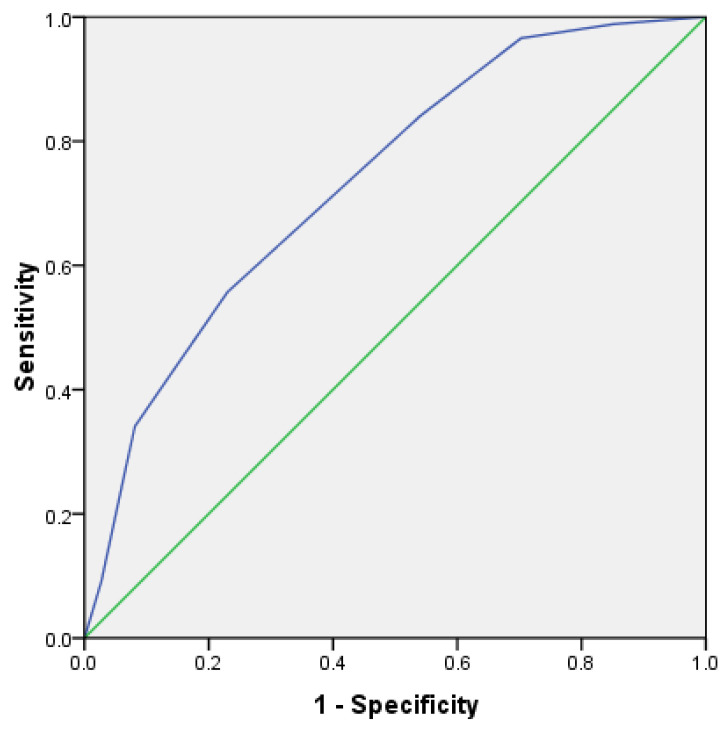
ROC curve for predicting survival risk of HLH (validation group).

In order to further verify the prediction efficiency of HLH scoring system, the ROC curve was analyzed. In the test group, the area under the ROC curve of 162 cases was 0.792, *p* < 0.001, and the 95% confidence interval was 0.723~0.861 (Figure 8). In addition, in the validation group, the area under the ROC curve of 162 cases was 0.736, *p* < 0.001, and the 95% confidence interval was 0.660~0.813 (Figure 9). This shows that it has good prediction ability.

## 4. Discussion

HLH includes two types: primary and secondary, but both primary and secondary include the activation of immune tissue cells in a superimposed state and the out-of-control regulation of the immune system. If not blocked, it can lead to continuous proliferation and activation coupled with blocked apoptosis, resulting in a high level of cytokines and a cytokine storm [5]. At present, the prognostic factors and survival time of HLH are uncertain, but understanding the adverse factors related to disease progression and prognosis is of great significance for the evaluation of the disease, the rational formulation of a treatment plan, and the improvement of the prognosis and survival rate of patients.

At present, there is no report on the prognostic scoring system of HLH in the literature at home or abroad. It is necessary to establish a widely accepted and adopted scoring system. Univariate analysis showed that OS was significantly correlated with old age (>60.0 year), male sex, lymphocytopenia (≤0.5 × 10^9^/L), thrombocytopenia (≤20 × 10^9^/L), coagulation dysfunction (APTT > 36.0 s, PT >13.5 s), urea abnormality ( > 8.2 mmol/L), LDH abnormality (>1000.0 U/L), ferritin abnormality (>10,000.0 μg/L), and CRP abnormality (>100.0 mg/L). Multivariate analysis indicated that male sex, coagulation dysfunction, abnormal LDH, and abnormal CRP were independent prognostic indicators of declined OS, which was consistent with previous research [6,7,8,9,10].

Shunichi et al. [11] reported that 116 patients with autoimmune-associated HLH, malesex (*p* < 0.01, HR = 6.47, 95% CI: 2.06~30.39) was identified as the factors associated with mortality. Coburn et al. [12] reported an incidence for systematically characterize HLH in moderate-to-severe inflammatory bowel disease. Additionally, found that HLH occurred more often in males (70.0%). Risk factors may include male sex, presence of Crohn’s disease, and induction phase of treatment.

Coagulation disorders are common during HLH and play a key role both in the global severity of the disease and in the occurrence of hemorrhagic complications [13]. Coagulation disorders confer a higher risk of bleeding, and this complication can be severe. Raised D-dimer levels and coagulation disorders are also reported in 50% of the cases, and nearly half of the patients fulfill disseminated intravascular coagulation (DIC) criteria [14,15,16]. In a retrospective study, 16 of 29 patients (55%) with lymphoma-related HLH had DIC [17]. Coagulation impairment is strongly correlated to the prognosis in patients with HLH [13]. Chen et al. [18] put forward that prolonged APTT > 44.35 s is a strong predictive factor for mortality. Multivariate Cox regression analysis demonstrated that APTT (*p* = 0.045, HR = 1.03, 95% Cl: 1.00~1.10) was an independent risk factor for mortality. DIC caused by coagulation dysfunction is also one of the main causes of HLH death [13,19,20]. Coagulation disorders are often related to severe systemic inflammation, DIC, and coagulation factor defects caused by liver failure. A large number of IFN cytokines such asγ, TNF, and IL-1 release activate cytotoxic T cells and macrophages, make them proliferate and activate in large numbers, produce hypercytokinemia, enhance macrophage phagocytosis, and cause coagulation disorders [21].

LDH could sensitively and comprehensively reflect the organ index of tissue damage. Clinical risk factors related to HLH included maximum LDH [22]. A second case series by Leow et al. [23] described a cohort of pediatric patients with HLH admitted to the cardiac ICU and assessed for poor prognostic factors and mortality. Patients with a higher median peak serum LDH levels were associated with higher mortality. Furthermore, elevated LDH was demonstrated to be a poor prognostic factor for survival in lymphoma associated hemophilus syndrome (LAHS). Jia et al. [20] put forward that univariate analysis showed that patients of NK/T-cell lymphoma associated hemophilus syndrome (NK/T-LAHS) with LDH > 1000 U/L (*p* = 0.048) and DIC (*p* = 0.004) had shorter survival time. Other studies also demonstrated that elevation of LDH was associated with unfavorable outcomes in patients with NK/T-LAHS [8,9,24,25]. Li et al. [9] reported that the risk factors for NK/T-LAHS was elevated LDH level (> 314 U/L) (*p* = 0.038, HR = 6.293, 95%Cl: 1.108~35.735). However, the optimal value of LDH serving as a risk factor in LAHS was unclear. Different studies show that 1000 U/L or 2000 U/L was the threshold value for 10 prognoses [20,26,27]. Zhang et al. [28] noticed that a four times elevation of LDH (>1000 U/L) predicted poor prognosis in patients with NK/T-LAHS. We inferred that the prominent elevation of LDH might be a direct consequence of the cytokine storm and hyperinflammation.

The prognosis of Epstein–Barr-virus-associated hemophilus syndrome (EBV-HLH) patients was significantly correlated with CRP [29]. Bozkurt et al. [30] showed that CRP was detected as mortality predictors in the univariate analysis. Fukaya et al. [31] multivariate analysis showed that the presence of infections and CRP level (>50 mg/L) on HLH related with poor prognosis, thus high CRP level may relate to infection rather than to HLH itself. Taking these studies and our study together, infections seem to be the common risk factor in adult HLH patients. Interestingly, CRP also showed correlations with ferritin values which might indicate that these inflammatory parameters are part of the cytokine pattern of HLH [32].

In our study, the four indexes were assigned one by one according to their weight in Cox regression, and a more accurate clinical prediction equation was established. According to the score, the patients were divided into low-risk, medium-risk, and high-risk groups. The 5-year OS of the low-risk group was 71.7%, that of the medium-risk group was 41.8%, and that of the high-risk group was 12.8%. There was significant difference in survival among the low-risk, medium-risk, and high-risk groups. In addition, in clinical practice, we further verified the efficacy of the HLH prognosis scoring system in HLH patients. It was found that the 2-year OS of patients in the low-risk, medium-risk, and high-risk groups were 88.0%, 45.1%, and 16.7%, respectively. This study also has some limitations. First, the risk scoring model needs to be further verified in prospective studies. Secondly, due to the large difference of adjuvant therapy in patients, it is not included in the analysis of influencing factors, and its impact on the prognosis of HLH is still unclear. This work needs to be further carried out.

In conclusion, male sex, APTT > 36 s, LDH > 1000 U/L and CRP > 100 mg/L are risk factors for the prognosis of HLH patients. The prognostic scoring system established in this study can be used to predict the long-term survival of HLH patients.

## Figures and Tables

**Table 1:** Characteristics of the patients investigated.

Variable	(324)
Age (year, (M))	51 (18–90)
>60 years of age (%)	105 (32.4)
Male (%)	188 (58.0)
Female (%)	136 (41.9)
T > 40.0 °C (%)	68 (20.9)
WBC ≤ 4.0 × 10^9^/L (%)	263 (81.2)
N ≤ 0.5 × 10^9^/L (%)	75 (23.1)
L ≤ 0.5 × 10^9^/L (%)	198 (61.1)
Hb ≤ 60 g/L (%)	82 (25.3)
Plt ≤ 20 × 10^9^/L (%)	128 (39.5)
Fib ≤ 1.5 g/L (%)	217 (66.9)
APTT > 36 s (%)	239 (73.8)
PT > 13.5 s (%)	201 (62.0)
D-Dimer > 700 μg/L (%)	283 (87.3)
ALB ≤ 30 g/L (%)	246 (75.9)
GLB ≤ 20 g/L (%)	190 (58.6)
ALT > 40 U/L (%)	255 (78.7)
AST > 50 U/L (%)	262 (80.9)
TB > 21 umol/L (%)	183 (56.5)
DB > 10 umol/L (%)	187 (57.7)
IB > 14 umol/L (%)	142 (43.8)
Cr abnormal (%)	208 (64.2)
Urea > 8.2 mmol/L (%)	164 (50.6)
TG > 3 mmol/L (%)	157 (48.5)
LDH > 1000 U/L (%)	141 (43.5)
Ferritin > 10,000 μg/L (%)	137 (42.3)
CRP > 100 mg/L (%)	76 (23.5)
Pneumonia (%)	160 (49.4)
Splenomegaly (%)	226 (69.8)
Hepatomegaly (%)	48 (14.8)
Lymphadenopathy (%)	91 (28.1)
Primary disease	
Tumour (%)	144 (44.4)
Connective tissue disease (%)	17 (5.2)
Infected (%)	86 (26.5)
Unknown etiology (%)	77 (23.9)
Death (%)	181 (55.9)

Abbreviations: T = temperature; WBC = white blood cell; N = neutrophils; L = lymphocyte; Hb = hemoglobin; Plt = platelet; Fib = fibrinogen; PT = prothrombin time; APTT = activated partial thromboplastin time; ALB = albumin; GLB= globulin; ALT = alanine transaminase; AST = aspartate amino transferase; TB = total bilirubin; DB = direct bilirubin; IB = Indirect bilirubin; Cr = creatinine; Urea= blood urea; TG = triglyceride; LDH = lactate dehydrogenase; CRP = C-reactive protein.

**Table 2:** Comparison of general data between test group and validation group.

Variable	Test Group (162)	Validation Group (162)	*p*
Age (year, (M))	54(18–88)	49(18–90)	
>60 years of age	55 (33.9)	50 (30.9)	0.553
Male (%)	97 (59.9)	91 (56.2)	0.499
Female (%)	65 (40.1)	71 (43.8)	0.499
T > 40.0 ℃ (%)	32 (19.8)	36 (22.2)	0.585
WBC ≤ 4.0 × 10^9^/L (%)	135 (83.3)	128 (79.0)	0.320
N ≤ 0.5 × 10^9^/L (%)	42 (25.9)	33 (20.4)	0.236
L ≤ 0.5 × 10^9^/L (%)	95 (58.6)	103 (63.6)	0.362
Hb ≤ 60 g/L (%)	42 (25.9)	40 (24.7)	0.798
Plt ≤ 20 × 10^9^/L (%)	54 (33.3)	74 (45.7)	0.023
Fib ≤ 1.5 g/L (%)	103 (63.6)	114 (70.4)	0.194
APTT > 36 s (%)	118 (72.8)	121 (74.7)	0.705
PT > 13.5 s (%)	110 (67.9)	91 (56.2)	0.030
D-Dimer > 700 μg/L (%)	137 (84.6)	146 (90.1)	0.133
ALB ≤ 30 g/L (%)	120 (74.1)	126 (77.8)	0.436
GLB ≤ 20 g/L (%)	91 (56.2)	99 (61.1)	0.367
ALT > 40 U/L (%)	129 (79.6)	126 (77.8)	0.684
AST > 50 U/L (%)	129 (79.6)	133 (82.1)	0.572
TB > 21 umol/L (%)	82 (50.6)	101 (62.3)	0.033
DB > 10 umol/L (%)	89 (54.9)	98 (60.5)	0.311
IB > 14 umol/L (%)	65 (40.1)	77 (47.5)	0.179
Cr abnormal (%)	106 (65.4)	107 (66.0)	0.907
Urea > 8.2 mmol/L (%)	76 (46.9)	88 (54.3)	0.182
TG > 3 mmol/L (%)	87 (53.7)	70 (43.2)	0.059
LDH > 1000 U/L (%)	70 (43.2)	71 (43.8)	0.911
Ferritin > 10,000 μg/L (%)	74 (45.7)	63 (38.9)	0.216
CRP > 100 mg/L (%)	38 (23.5)	38 (23.5)	1.000
Pneumonia (%)	77 (47.5)	83 (51.2)	0.505
Splenomegaly (%)	111 (68.5)	115 (70.9)	0.629
Hepatomegaly (%)	26 (16.0)	22 (13.6)	0.532
Lymphadenopathy (%)	41 (25.3)	50 (30.9)	0.266
Primary disease			
Tumour (%)	60 (37.0)	85 (52.5)	0.005
Connective tissue disease (%)	8 (4.9)	9 (5.6)	0.617
Infected (%)	65 (40.1)	41 (25.3)	0.004
Unknown etiology (%)	29 (17.9)	27 (16.7)	0.769

**Table 3:** Common data of the patients in survival and dead group.

Variable	Survival Group (69)	Death Group (93)	*p*
Age(year, (M))	42(18–81)	57(18–88)	
>60 years of age	14(20.3)	41(44.1)	0.002
Male (%)	33(47.8)	64(68.8)	0.007
Female (%)	36(52.2)	29(31.2)	0.007
T > 40.0 °C (%)	11(15.9)	21(22.6)	0.294
WBC ≤ 4.0 × 10^9^/L (%)	54(78.3)	81(87.1)	0.136
N ≤ 0.5 × 10^9^/L (%)	14(20.3)	28(30.1)	0.159
L ≤ 0.5 × 10^9^/L (%)	31(44.9)	64(68.8)	0.002
Hb ≤ 60 g/L (%)	15(21.7)	27(29.0)	0.295
Plt ≤ 20 × 10^9^/L (%)	13(18.8)	41(44.1)	0.001
Fib ≤ 1.5 g/L (%)	37(53.6)	66(70.9)	<0.001
APTT > 36 s (%)	38(55.1)	80(86.0)	<0.001
PT > 13.5 s (%)	37(53.6)	73(78.5)	0.001
D-Dimer > 700 μg/L (%)	58(84.1)	79(84.9)	0.877
ALB ≤ 30 g/L (%)	49(71.0)	71(76.3)	0.444
GLB ≤ 20 g/L (%)	34(49.3)	57(61.3)	0.127
ALT > 40 U/L (%)	56(81.2)	73(78.5)	0.677
AST > 50 U/L (%)	52(75.4)	77(82.8)	0.245
TB > 21 umol/L (%)	34(49.3)	48(51.6)	0.769
DB > 10 umol/L (%)	34(49.3)	55(59.1)	0.212
IB > 14 umol/L (%)	23(33.3)	42(45.2)	0.129
Cr abnormal (%)	43(62.3)	61(65.6)	0.667
Urea > 8.2 mmol/L (%)	24(34.8)	52(55.9)	0.008
TG > 3 mmol/L (%)	33(47.8)	54(58.1)	0.253
LDH > 1000 U/L (%)	16(23.2)	54(58.1)	<0.001
Ferritin > 10,000 μg/L (%)	24(34.8)	50(53.8)	0.016
CRP > 100 mg/L (%)	9(13.0)	29(31.2)	0.007
Pneumonia (%)	31(44.9)	46(49.5)	0.568
splenomegaly (%)	43(62.3)	48(51.6)	0.174
Hepatomegaly (%)	9(13.0)	17(18.3)	0.369
Lymphadenopathy (%)	17(24.6)	24(25.8)	0.866
Primary disease			
Tumour (%)	24(34.8)	36(38.7)	0.609
Connective tissue disease (%)	9(13.0)	1(1.1)	0.004
Infected (%)	23(33.3)	26(27.9)	0.461
Unknown etiology (%)	13(18.9)	30(32.3)	0.056

Abbreviations: T = temperature; WBC = white blood cell; N = neutrophils; L = lymphocyte; Hb = hemoglobin; Plt = platelet; Fib = fibrinogen; PT = prothrombin time; APTT = activated partial thromboplastin time; ALB = albumin; GLB= globulin; ALT = alanine transaminase; AST = aspartate amino transferase; TB = total bilirubin; DB = direct bilirubin; IB = Indirect bilirubin; Cr = creatinine; Urea= blood urea; TG = triglyceride; LDH = lactate dehydrogenase; CRP = C-reactive protein.

**Table 4:** COX proportional hazards model analysis.

Variable	Univariate Variable			Multivariate Variables		
	OR	95%Cl	*p*	OR	95%Cl	*p*
>60 years of age	1.953	1.291–2.955	0.002	1.231	0.778–1.946	0.375
Male	1.692	1.090–2.627	0.019	1.778	1.111–2.844	0.016
L ≤ 0.5 × 10^9^/L	1.801	1.160–2.797	0.009	1.469	0.914–2.361	0.112
Plt ≤ 20 × 10^9^/L	2.059	1.362–3.114	0.001	1.543	0.963–2.471	0.071
Fib ≤ 1.5 g/L	1.541	0.983–2.415	0.059			
APTT > 36 s	2.945	1.634–5.306	0.000	2.003	1.055–3.803	0.034
PT > 13.5 s	1.952	1.189–3.207	0.008	1.013	0.579–1.773	0.963
Urea > 8.2 mmol/L	1.631	1.081–2.460	0.020	1.066	0.679–1.673	0.783
LDH > 1000 U/L	2.420	1.593–3.675	0.000	2.046	1.256–3.333	0.004
Ferritin > 10,000 μg/L	1.584	1.052–2.384	0.028	1.113	0.705–1.756	0.645
CRP > 100 mg/L	1.811	1.164–2.820	0.009	1.657	1.038–2.646	0.034
Connective tissue disease	0.154	0.021–1.104	0.063			

Abbreviations: L = lymphocyte; Plt = platelet; Fib = fibrinogen; PT = prothrombin time; APTT = activated partial thromboplastin time; Urea= blood urea; LDH = lactate dehydrogenase; CRP = C-reactive protein.

**Table 5:** Prediction Model.

Variable		Score
Gender	Male	1
	Female	0
APTT	≤36 s	0
	>36 s	2
LDH	≤1000 U/L	0
	>1000 U/L	2
CRP	≤100 mg/L	0
	>100 mg/L	1
Risk Group		Total Score
Low-risk group		0–1
Moderate-risk group		2–4
Height-risk group		5–6

Abbreviations: APTT = activated partial thromboplastin time; LDH = lactate dehydrogenase; CRP = C-reactive protein.

## Data Availability

Supplementary data to this article can be found online at https://figshare.com/s/a1bffb14f5291fbfbbe2.

## Supplementary Materials

The following supporting information can be downloaded at https://figshare.com/s/a1bffb14f5291fbfbbe2.
